# The factors associated with impulse control behaviors in Parkinson's disease: A 2‐year longitudinal retrospective cohort study

**DOI:** 10.1002/brb3.1036

**Published:** 2018-06-29

**Authors:** Tomoya Kon, Tatsuya Ueno, Rie Haga, Masahiko Tomiyama

**Affiliations:** ^1^ Department of Neurology Aomori Prefectural Central Hospital Aomori Japan; ^2^ Department of Neuropathology Graduate School of Medicine Hirosaki University Hirosaki Japan

**Keywords:** dopamine agonist, dopamine dysregulation syndrome, impulse control behavior, impulse control disorder, Parkinson's disease

## Abstract

**Introduction:**

Impulse control behaviors (ICBs) are impulsive–compulsive behaviors often associated with dopamine replacement therapy in Parkinson's disease (PD). Although remission can occur in ICB, only four reports on the ratio of remission and the persistence of ICB have been published, and the associated factors with ICB remission or persistence have been little known. Therefore, we conducted a longitudinal assessment of the remission, persistence, and development of ICB and those associated factors in patients with PD.

**Methods:**

We retrospectively investigated a PD database at Aomori Prefectural Central Hospital, Japan. One hundred and forty‐eight patients with PD who could be followed up for 2 years were enrolled. ICB was assessed using the Questionnaire for Impulsive–Compulsive Disorders in Parkinson's disease. Motor severity (Hoehn and Yahr scale and United Parkinson's Disease Rating Scale), cognitive function (Mini–Mental State Examination), and other clinical variables (sex, age, onset age, disease duration, olfactory dysfunction, and dyskinesia) and medications used to treat PD were assessed. Univariate analyses were performed.

**Results:**

Seven patients were excluded because of the exclusion criteria, and 141 patients were analyzed. Thirty patients (21.3%) had ICB at baseline, and these patients also had significantly higher use of pergolide. The ICB remission rate was 60%, the ICB persistence ratio was 40%, and the ICB development ratio was 12.6% over 2 years. Statistically, younger age and pergolide use were associated with ICB persistence. Being male, having dyskinesia, and rotigotine, entacapone, zonisamide, and istradefylline use were associated with ICB development.

**Conclusion:**

This study suggests that younger age and pergolide use may be the new associated factors with ICB persistence and that entacapone, zonisamide, and istradefylline use may be associated with the development of ICB. Drug profiles and medication practices in Japan may explain the association of these factors with ICB.

## 1 INTRODUCTION

Impulse control disorder (ICD) generally refers to four major disorders: pathological gambling, compulsive sexual behavior, compulsive buying, and binge eating. Impulse control behaviors (ICBs) consist of ICDs and a number of closely related behaviors, including punding, hobbyism, walkabout, and dopamine dysregulation syndrome (DDS) (Zurowski & O'Brien, 2015). The prevalence of ICD or ICB has been reported to be 3.5%–58.3% in patients with Parkinson's disease (PD) (Antonini et al., 2017; Callesen, Weintraub, Damholdt, & Moller, 2014; Erga, Alves, Larsen, Tysnes, & Pedersen, 2017; Fan, Ding, Ma, & Chan, 2009; Rodriguez‐Violante, Gonzalez‐Latapi, Cervantes‐Arriaga, Camacho‐Ordonez, & Weintraub, 2014; Vela et al., 2016). ICB is an important nonmotor complication in patients with PD because it is associated with functional impairment, decreased quality of life and increased caregiver burden (Weintraub, David, Evans, Grant, & Stacy, 2015). Previous studies have shown that dopamine agonist (DA) use is the most consistent predictor of ICB (Weintraub, Koester, et al., 2010; Weintraub, et al., 2015; Zurowski & O'Brien, 2015). ICB is also independently associated with younger age, younger age at PD onset, previous ICB history, family history of gambling disorders, concurrent cigarette smoking, unmarried status, depression, anxiety, alcohol use, novelty‐seeking traits, higher DA dosage, combination DA and L‐DOPA therapy, and higher L‐dopa doses (Weintraub, Koester, et al., 2010; Weintraub, et al., 2015; Zurowski & O'Brien, 2015). There are conflicting data about whether amantadine and deep brain stimulation may be a treatment or a potential risk factor for ICB (Cera et al., 2014; Weintraub, Sohr, et al., 2010; Weintraub et al., 2015; Zurowski & O'Brien, 2015).

A number of cross‐sectional studies of ICB in PD have been conducted, but longitudinal assessments have been less reported (Antonini et al., 2017; Avila, Cardona, Martin‐Baranera, Bello, & Sastre, 2011; Bastiaens, Dorfman, Christos, & Nirenberg, 2013; Mamikonyan et al., 2008; Siri et al., 2015; Smith, Xie, & Weintraub, 2016; Sohtaoglu, Demiray, Kenangil, Ozekmekci, & Erginoz, 2010) and little is known about the course or change in ICB according to the type of medication. ICBs are not permanent symptoms and can go into remission (Avila et al., 2011; Mamikonyan et al., 2008; Siri et al., 2015; Sohtaoglu et al., 2010). In previous studies, the ICB remission ratio in PD was reported to be 40% during 3.5 years of follow‐up period (Siri et al., 2015) to 73.3% over 29.2 months (Mamikonyan et al., 2008). The factors associated with ICB remission were reduction in DA use (Avila et al., 2011), lower DA dosage (Mamikonyan et al., 2008; Sohtaoglu et al., 2010), and better working memory performance (Bastiaens et al., 2013). However, the reduction or discontinuation of DA may induce DA withdrawal symptoms and worsen motor symptoms, meaning that formulating a treatment strategy for ICB is problematic (Weintraub et al., 2015; Zurowski & O'Brien, 2015). Furthermore, little is known about the factors regarding ICB remission and persistence. Therefore, we conducted a longitudinal case–control study of the remission, persistence, and development of ICB and its associated factors, particularly in relation to medication in patients with PD.

## 2 MATERIAL AND METHODS

### 2.1 Study design and participants

We retrospectively investigated sequential outpatients with PD using a computerized medical database of the movement disorder clinic at Aomori Prefectural Central Hospital, Aomori, which is a regional tertiary center located in northern territory of Japan. The recruitment period was from April 2014 to May 2015. The inclusion criteria were as follows: patients diagnosed with PD (hereinafter referred to as PD patients) according to the UK Brain Bank criteria (Hughes, Daniel, Kilford, & Lees, 1992) and patients who could be followed up over a 2‐year observational period. The exclusion criteria were as follows: patients with dementia, patients with any missing data, and the “exclusion criteria for PD” which was described in the UK Brain Bank criteria (Hughes et al., 1992). Dementia was diagnosed if the patient had a Mini–Mental State Examination MMSE score ≦23 (Holsinger, Deveau, Boustani, & Williams, 2007) at baseline.

The Questionnaire for Impulsive–Compulsive Disorders in Parkinson's disease (QUIP) is a self‐administered questionnaire specifically assessing ICB (pathological gambling, sexual behavior, compulsive buying, binge eating, punding, hobbyism, walkabout, and DDS) in PD (Weintraub et al., 2009). All patients completed a validated Japanese version of the QUIP short form (Tanaka, Wada‐Isoe, Nakashita, Yamamoto, & Nakashima, 2013) at each visit because we administer this as a standard practice at our institution. When the patient had at least one “yes” response, we interviewed them and their caregivers to confirm whether those behaviors were ICB by the established diagnostic criteria (Voon & Fox, 2007). We defined ICB as positive in cases with both at least one “yes” in QUIP and fulfilling the diagnostic criteria. When the patient answered “no” to ICB symptoms, we confirmed this by self‐report and by asking their caregivers. We defined ICB as being in remission when patients who had ICB at baseline screened negative for any QUIP at the 2‐year follow‐up. We then classified patients into “ICB persister” (ICB at baseline and follow‐up), “ICB remitter” (ICB at baseline but not follow‐up), and “ICB developer” (no ICB at baseline, but ICB at follow‐up) groups. The following assessments were also performed by the movement disorder specialists at each visit: Hoehn and Yahr scale, United Parkinson's Disease Rating Scale, MMSE, olfactory dysfunction and dyskinesia. The presence of olfactory dysfunction was assessed only by interview, not by specific odor tests. The presence of dyskinesia was evaluated with history taking and neurological examination. The types and doses of PD medication prior to the visit were recorded at each visit. L‐DOPA equivalent dose (LED) was calculated using a formula reported by Tomlinson et al. (2010). The differences in medication dosage between baseline and 2‐year follow‐up are expressed as ΔLED.

This study was performed in accordance with the Declaration of Helsinki and was approved by the ethics committee of Aomori Prefectural Central Hospital. Written informed consent for participation in the study was not obtained from the patients because the study was retrospective in nature and the data were analyzed anonymously.

### 2.2 Statistical analysis

Demographic data were compared between ICB‐positive and ICB‐negative patients using chi‐square tests for categorical variables and Mann–Whitney *U* tests for continuous variables. Multivariate analysis was not able to be performed because of the small sample size. Statistical analysis was performed using the software program Excel‐toukei (BellCurve; Tokyo, Japan). Data are shown as median values and interquartile ratios. All analyses were exploratory in nature, and *p* values of <0.01 were defined as significant to minimize the effects from multiple comparisons based on the concept of Bonferroni correction.

## 3 RESULTS

**Figure 1:**
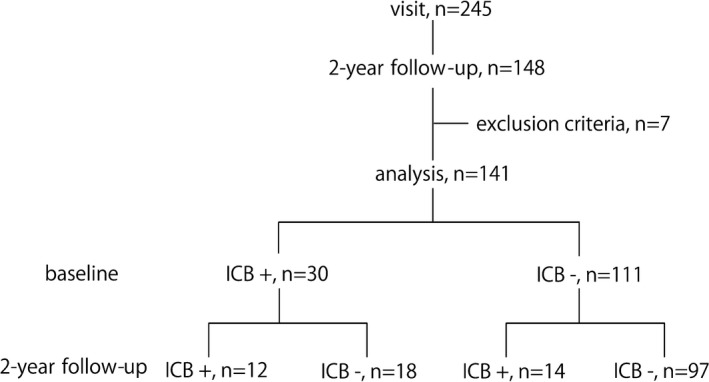
Study flow. Two hundred and forty‐five patients visited our movement disorder clinic during recruitment period. One hundred and forty‐eight patients were followed up over a 2‐year observational period. Seven patients were excluded by the exclusion criteria. Finally, 141 patients were analyzed. At baseline, 30 patients had impulse control behaviors (ICB) and 111 patients did not have ICB. At the 2‐year follow‐up of the patients with ICB at baseline (*n* = 30), 12 had persistent ICB while 18 had ICB in remission, and of the patients without ICB at baseline (*n* = 111), 14 developed ICB while 97 patients did not develop ICB

Two hundred and forty‐five PD patients visited our PD clinic during the study period. Of these, 148 patients were followed up for 2 years, seven patients were excluded by the exclusion criteria, and 141 patients were enrolled in the study (Figure 1).

### 3.1 Baseline demographic data

The patients' demographic characteristics at baseline are presented in Table 1. Overall, 21.3% (30/141) of patients had at least one ICB. L‐DOPA was used by 95.0% (134/141), and DA was used by 46.8% (66/141) in our cohorts. Pergolide use was higher in patients with ICB than those without ICB (23% vs. 5.4%, *p* = 0.003). No other characteristics were significantly different. The prevalences of QUIP items at baseline are presented in Table 2. Hobbyism was the most frequent ICB symptom (43.3%), and no one had any symptoms of walkabout. The numbers of QUIP module positivity were as follows: single, 53.3% and multiple, 46.7% (Table 2).

**Table 1:** Patient demographics at baseline

	ICB+, *n* = 30	ICB−, *n* = 111	*p*
Sex, male (%)	14 (46.7)	37 (33.3)	0.178
Age, median (IQR)	68 (63.3–75.8)	71 (64.5–75)	0.542
Onset age, year, median (IQR)	62 (53.5–68.8)	65 (59–70)	0.25
Disease duration, year, median (IQR)	6.5 (10–26)	5 (3–9)	0.091
Olfactory dysfunction, *n* (%)	17 (56.7)	40 (36)	0.041
Dyskinesia, *n* (%)	8 (26.7)	23 (20.7)	0.485
Deep brain stimulation treatment, *n* (%)	0 (0)	3 (2.7)	0.363
MMSE score, median (IQR)	28 (25–29)	27 (25–29.5)	0.897
HY stage, median (IQR)	2.5 (2–3)	2 (2–3)	0.313
UPDRS part III, median (IQR)	14 (10–26)	19 (12–28.5)	0.174
Medication			
L‐DOPA use, *n* (%)	28 (93.3)	106 (95.5)	0.629
L‐DOPA use, monotherapy, *n* (%)	8 (26.7)	31 (27.9)	0.891
DA use, *n* (%)	20 (66.7)	46 (41.4)	0.014
Pramipexole IR use, *n* (%)	4 (13.3)	5 (4.5)	0.079
Pramipexole ER use, *n* (%)	4 (13.3)	19 (17.1)	0.619
Ropinirole CR use, *n* (%)	3 (10)	17 (15.3)	0.459
Rotigotine use, *n* (%)	0 (0)	7 (6.3)	0.158
Pergolide use, *n* (%)	7 (23)	6 (5.4)	**0.003**
Amantadine use, *n* (%)	12 (40)	23 (20.7)	0.033
Selegiline use, *n* (%)	6 (20)	16 (14.4)	0.454
Entacapone use, *n* (%)	5 (16.7)	13 (11.7)	0.471
Zonisamide use, *n* (%)	9 (30)	19 (17.1)	0.117
Istradefylline use, *n* (%)	1 (3.3)	6 (5.4)	0.643
LED‐total mg, median (IQR)	657 (450–845)	530 (378–782)	0.182
LED‐L‐DOPA mg, median (IQR)	450 (300–600)	400 (300–525)	0.329
LED‐DA mg, median (IQR)	125 (0–197)	38 (0–240)	0.815
LED‐other PD drugs mg, median (IQR)	12.5 (0–150)	0 (0–100)	0.110

*Note*

CR: controlled release; DA: dopamine agonist; ER: extended release; HY: Hoehn and Yahr scale; ICB: impulse control behavior; IQR: interquartile range; IR: immediate release; LED: L‐DOPA equivalent dose; MMSE: Mini–Mental State Examination; UPDRS: United Parkinson's Disease Rating Scale.

Bold indicates *p* <0.01.

**Table 2:** Types and numbers of ICB

	Baseline	2‐year follow‐up		*p* (ICB persister vs. ICB developer)
	ICB+, *n* = 30	ICB persister, *n* = 12	ICB developer, *n* = 14	
ICB subtypes, *n* (%)				
Pathological gambling	7 (23.3)	3 (25)	1 (7.1)	0.453
Compulsive sexual behavior	5 (16.7)	2 (16.7)	1 (7.1)	0.750
Compulsive buying	6 (20)	4 (33.3)	2 (14.3)	0.517
Binge eating	6 (20)	3 (16.7)	4 (28.6)	0.979
Punding	6 (20)	6 (50)	5 (35.7)	0.763
Hobbyism	13 (43.3)	5 (41.7)	6 (42.9)	0.998
Walkabout	0	0	0	
Dopamine dysregulation syndrome	8 (26.7)	5 (41.7)	2 (14.3)	0.292
Number of QUIP module, *n* (%)				
Single	16 (53.3)	6 (50)	8 (57.1)	0.934
Multiple	14 (46.7)	6 (50)	6 (42.9)	0.936

*Note*

ICB: impulse control behavior; QUIP: Questionnaire for Impulsive–Compulsive in Parkinson's Disease.

### 3.2 Two‐year follow‐up

After 2 years of follow‐up, 60% (18/30) of patients with ICB at baseline had ICB in remission, 40% (12/30) of those had persistent ICB, and 12.6% (14/111) had developed ICB. At the 2‐year follow‐up, the prevalence of ICB was significantly higher in patients with ICB at baseline than those without ICB at the baseline (*p* < 0.001). No patients had initiated DBS therapy during the 2 years.

The characteristics of patients at 2‐year follow‐up are shown in Table 3. Patients identified as “ICB persisters” were significantly younger (*p* = 0.005) and had more pergolide use (*p* = 0.005) than “ICB remitter” patients. No other variables were statistically significantly different between these groups.

**Table 3:** Characteristics of patients at the 2‐year follow‐up

	ICB+ at baseline			ICB− at baseline		
	ICB persister, *n* = 12	ICB remitter, *n* = 18	*p*	ICB developer, *n* = 14	ICB−, *n* = 97	*p*
Demographics						
Sex, male (%)	5 (41.7)	9 (50)	0.654	9 (64.3)	28 (28.9)	**0.009**
Age, median (IQR)	66 (56.8–69.8)	77 (69–80.8)	**0.005**	73.5 (58.8–75)	74 (67–78)	0.221
Onset age, year, median (IQR)	57 (19–62)	66.5 (57.3–69)	0.037	60 (50.3–65.5)	66 (59–70)	0.057
Disease duration, year, median (IQR)	7 (5.8–9)	11 (7.3–12)	0.065	8.5 (7.3–11)	8 (5–11)	0.416
Olfactory dysfunction, *n* (%)	7 (58.3)	10 (55.6)	0.880	5 (35.7)	35 (36.1)	0.979
RBD, *n* (%)	6 (50)	7 (38.9)	0.547	7 (50)	40 (41.2)	0.535
Dyskinesia, *n* (%)	4 (33.3)	6 (33.3)	1	7 (50)	17 (17.5)	**0.006**
MMSE score, median (IQR)	28.5 (27.8–30)	25 (22.3–29.3)	0.075	26 (22.3–28.8)	28 (25–30)	0.184
HY, median (IQR)	2.3 (2–3)	3 (2.5–3.5)	0.052	2 (2–3)	2.5 (2–3)	0.512
UPDRS part 3, median (IQR)	14 (8.5–22)	24.5 (16.3–29)	0.082	16 (10–21.3)	19 (11–31)	0.386
Medication						
L‐DOPA use, *n* (%)	12 (100)	18 (100)	1	14 (100)	95 (97.9)	0.588
L‐DOPA use, monotherapy, *n* (%)	1 (8.3)	5 (27.8)	0.192	1 (7.1)	19 (19.6)	0.257
DA use, *n* (%)	10 (83.3)	9 (50)	0.063	12 (85.7)	50 (51.5)	0.016
Pramipexole IR use, *n* (%)	0	2 (11.1)	0.232	1 (7.1)	3 (3.1)	0.447
Pramipexole ER use, *n* (%)	1 (8.3)	3 (16.7)	0.511	2 (14.3)	12 (12.4)	0.840
Ropinirole CR use, *n* (%)	3 (25)	0 (0)	0.025	3 (21.4)	17 (17.5)	0.723
Rotigotine use, *n* (%)	1 (8.3)	2 (11.1)	0.804	4 (28.6)	11 (11.3)	0.078
Pergolide use, *n* (%)	6 (50)	1 (5.6)	**0.005**	2 (14.3)	7 (7.2)	0.366
Amantadine use, *n* (%)	4 (33.3)	5 (27.8)	0.745	1 (7.4)	20 (20.6)	0.229
Selegiline use, *n* (%)	4 (33.3)	4 (22.2)	0.500	5 (35.7)	23 (23.7)	0.252
Entacapone use, *n* (%)	6 (50)	5 (27.8)	0.216	4 (28.6)	14 (14.3)	0.180
Zonisamide use, *n* (%)	5 (41.7)	6 (33.3)	0.643	8 (57.1)	16 (16.5)	**<0.001**
Istradefylline use, *n* (%)	1 (8.3)	2 (11.1)	0.144	4 (28.6)	11 (11.3)	0.080
LED‐total mg, median (IQR)	782.5 (593.8–1,060)	740 (612.5–918.8)	0.966	788 (598–1,156)	610 (500–850)	0.099
LED‐L‐DOPA mg, median (IQR)	400 (300–625)	575 (450–600)	0.223	500 (363–588)	500 (300–600)	0.961
LED‐DA mg, median (IQR)	137.5 (125–245)	75 (0–275)	0.384	200 (150–300)	75 (0–280)	0.021
LED‐other PD medication, median (IQR)	195 (0–231)	77.5 (0–169)	0.289	37.5 (0–165)	0 (0–150)	0.480
ΔLED‐total mg, median (IQR)	70 (0–197.5)	70 (0–198)	0.896	12.5 (0–95)	100 (0–215)	0.183
ΔLED‐L‐DOPA mg, median (IQR)	0 (0–100)	25 (0–150)	0.555	0 (0–150)	0 (0–150)	0.3
ΔLED‐DA mg, median (IQR)	0 (0–46.3)	0 (0–0)	0.434	0 (0–28.8)	0 (0–5)	0.984

*Note*

CR: controlled release; DA: dopamine agonist; ER: extended release; ICB: impulse control behavior; IQR: interquartile range; IR: immediate release; LED: L‐DOPA equivalent dose; RBD: rapid eye movement sleep behavior disorder.

Bold indicates *p* <0.01.

Patients without ICB at baseline but who developed it over the 2 years of follow‐up significantly more likely to be male (*p* = 0.009), and have more like to have dyskinesia (*p* = 0.006), and zonisamide use (*p* < 0.001) than patients who had not developed ICB. Other variables did not differ between the two groups.

Punding and hobbyism were the most frequent ICBs, and no patient had symptoms of walkabout (Table 2). The types of ICB and the numbers of positive QUIP modules did not differ between ICB persister and ICB developer patients (Table 2).

Dose change medications during the 2‐year follow‐up are shown in Table 4. There were no differences in any aspects of PD medication between ICB persister and ICB remitter patients in the group with ICB at baseline. Of the group without ICB at baseline, 57.1% in the ICB developer group had DA treatment initiated or the dose increased, versus 28.9% of those without ICB. Rotigotine (*p* = 0.005), entacapone (*p* = 0.005), zonisamide (*p* < 0.001), and istradefylline (*p* = 0.001) were initiated or doses increased more frequently in ICB developer than those without ICB (Table 4). Of the group without ICB at baseline, 64.3% had their DA discontinued, or dose reduced in ICB developer group versus 15.5% in patients without ICB (*p* < 0.001) (Table 4). Pramipexole ER (*p* = 0.001) were discontinued or doses reduced more frequently in ICB developer than in patients without ICB (Table 4).

**Table 4:** Dose changes to medications during the 2‐year follow‐up

	ICB+ at baseline			ICB− at baseline		
	ICB persister, *n* = 12	ICB remitter, *n* = 18	*p*	ICB developer, *n* = 14	ICB−, *n* = 97	*p*
Initiated or dose increased medication, *n* (%)						
L‐DOPA	3 (25)	9 (50)	0.171	7 (50)	36 (37.1)	0.355
Dopamine agonist	3 (25)	4 (22.2)	0.860	8 (57.1)	28 (28.9)	0.035
Pramipexole IR	0	0		0	1 (1.0)	0.703
Pramipexole ER	0	1 (5.6)	0.406	0	5 (5.2)	0.385
Ropinirole CR	1 (8.3)	1 (5.6)	0.765	1 (7.1)	10 (10.3)	0.711
Rotigotine	1 (8.3)	1 (5.6)	0.765	5 (35.7)	9 (9.3)	**0.005**
Pergolide	1 (8.3)	1 (5.6)	0.765	2 (14.3)	3 (3.1)	0.059
Amantadine	0	1 (5.6)	0.406	0	7 (7.2)	0.299
Selegiline	2 (16.7)	1 (5.6)	0.320	4 (28.6)	14 (14.4)	0.180
Entacapone	4 (33.3)	2 (11.1)	0.136	5 (35.7)	9 (9.3)	**0.005**
Zonisamide	3 (25)	4 (22.2)	0.860	6 (42.9)	9 (9.3)	**<0.001**
Istradefylline	1 (8.3)	2 (11.1)	0.804	5 (35.7)	7 (7.2)	**0.001**
Droxidopa	0	1 (5.6)	0.406	0	4 (4.1)	0.439
Trihexyphenidyl	0	0	1	0	1 (1.0)	0.703
Discontinued or dose reduced medication, *n* (%)						
L‐DOPA	0	0		3 (21.4)	6 (6.2)	0.051
Dopamine agonist	0	0		9 (64.3)	15 (15.5)	**<0.001**
Pramipexole IR	0	2 (11.1)	0.232	0	0	
Pramipexole ER	0	0		3 (21.4)	2 (2.1)	**0.001**
Ropinirole CR	0	0		2 (14.3)	8 (8.2)	0.461
Rotigotine	0	0		1 (7.1)	2 (2.1)	0.273
Pergolide	0	0		1 (7.1)	2 (2.1)	0.273
Amantadine	2 (16.7)	0	0.073	3 (21.4)	5 (5.2)	0.028
Selegiline	0	1 (5.6)	0.406	0	1 (1.0)	0.703
Entacapone	0	1 (5.6)	0.406	0	3 (3.1)	0.505
Zonisamide	0	0		1 (7.1)	3 (3.1)	0.447
Istradefylline	1 (8.3)	0	0.213	0	2 (2.1)	0.588

*Note*

CR: controlled release; ER: extended release; ICB: impulse control behavior; IR: immediate release.

Bold indicates *p* <0.01.

## 4 DISCUSSION

We conducted longitudinal assessments of the remission, persistence and development of ICB, and the associated risk factors in a cohort study of over 140 PD patients. To our knowledge, this is the first report: (a) the ICB remission ratio using QUIP, (b) the potential association of ICB persistence with younger age and pergolide use, (c) the potential association of ICB development with zonisamide and istradefylline use, and (d) a longitudinal assessment from East Asia.

The ratio of ICB remission was 60% and that of development was 12.6% over the 2 years of our study. In previous studies, the ICB remission ratio in PD was found to range from 40% over a 3.5‐year follow‐up period by Siri et al. (2015) in Italy, 58.1% over 12.1 months by Avila et al. (2011) in Spain, 72.7% over 43.2 months by Sohtaoglu et al. (2010) in Turkey to 73.3% over 29.2 months by Mamikonyan et al. (2008) in the United States. Our remission ratio was similar to the report of Avila et al. (2011), higher than that of Siri et al. (2015), and lower than those of Sohtaoglu et al. (2010) and Mamikonyan et al. (2008). Although why the ICB remission ratio varies is very complex, we speculate that the differences may be explained by methodological factors, ethnic factors, environmental backgrounds, or the frequency of dopamine replacement therapy (DRT) (Chiang, Huang, Chen, & Wu, 2012; Tanaka et al., 2013).

Methodologically, other than the study of Siri et al. (2015), no other previous studies and the current study investigated patients retrospectively. And the screening tools to detect ICB were different. ICB was assessed by the Minnesota Impulse Disorder Interview (MIDI) by Mamikonyan et al. (2008). Diagnostic and Statistical Manual of Mental Disorders 4th edition (DSM‐IV) and criteria of their own by Sohtaoglu et al. (2010)), Voon and Fox's criteria by Avila et al. (2011) and Siri et al. (2015). The MIDI (Christenson et al., 1994) can be used to evaluate pathological gambling, sexual behavior, and compulsive buying; in addition to them, Voon and Fox's criteria include assessment of binge eating, punding, and DDS. The QUIP evaluates these ICB categories and is able to hobbyism and walkabout. Therefore, QUIP covers wider ICB categories than other criteria. The criteria with have narrower categories (MIDI, DSM‐IV) tend to reveal higher ICB remission ratios, whereas those of wider categories (Voon and Fox's criteria) seem to reveal lower ICB remission ratios than the current study. QUIP is becoming a standard screening tool to assess ICB in PD, which is used by Parkinson's Progression Markers Initiative (http://www.ppmi-info.org/) because it is comprehensive, self‐administered, and easy to complete (patients are able to complete the short form within 3 min) (Weintraub et al., 2009). It is also able to evaluate a wide range of ICB symptoms in one instrument. The disadvantage of QUIP is its low specificity but high sensitivity. In a direct comparison of QUIP and MIDI, the prevalence of ICB by QUIP has been 5% higher than MIDI (Antonini et al., 2017). Moreover, QUIP was positive in 20.3% in healthy subjects (Weintraub, Papay, & Siderowf, 2013). Therefore, to determine ICB diagnosis, other diagnostic criteria are needed. In our study, we adopted Voon and Fox's diagnostic criteria, when QUIP screening was positive.

In regard to ethnic factors, the ICD and ICB prevalences in Asian countries are relatively low by comparison with Western countries (Chiang et al., 2012; Tanaka et al., 2013; Wang, Wei, & Xiao, 2016). The prevalence of ICB in patients with PD using QUIP varies between 18.5% (Weintraub et al., 2013) to 58.3% (Vela et al., 2016) in Western countries, 35.0% in Malaysia (Lim et al., 2011), and 42.8% in India (Sharma et al., 2015). In our study, the prevalence of ICB at baseline was 21.3%, which is similar to a previous QUIP study in Japan (21.5%) (Tanaka et al., 2013). Medication practices, cost burdens, and health insurance have been suggested as potential factors with influencing the different prevalences (Fan et al., 2009). Genetic variants of serotonin receptors and polymorphisms for the dopaminergic, serotonergic, glutamate, and opioidergic systems have been related to the development of ICB in patients with PD (Comings & Blum, 2000; Ibanez, Blanco, Perez de Castro, Fernandez‐Piqueras, & Saiz‐Ruiz, 2003; Kraemmer et al., 2016; Le Foll, Gallo, Le Strat, Lu, & Gorwood, 2009; Lee, Jeon, Kim, & Park, 2012; Lee et al., 2009; Zainal Abidin et al., 2015). Thus, genetic variations in receptors, transporters, or enzymes of the catecholaminergic, serotonergic, glutamatergic, and opioid neurotransmission systems are potentially associated with ICD in PD (Jimenez‐Urbieta et al., 2015).

To consider environmental backgrounds, accessibility to gambling differs among countries, and casinos are not present in Japan. Hobbyism was the most frequent ICB symptom in our cohorts. The technological opportunities available to Japanese people today may lead to high rates of hobbyism (e.g., playing smartphone games or spending more time on the Internet, on surfing or on social networking services). We speculate that this could influence the prevalence of ICBs especially in younger patients.

Dopamine agonist was used by 46.8% of patients in our study (Table 1), which is relatively lower than the previous QUIP study in Japan (56%) (Tanaka et al., 2013). Proposed treatment strategies from the “Treatment Guideline for PD 2011” by the Japanese Society of Neurology (https://neurology-jp.org/guidelinem/parkinson.html) recommend that DA be the first‐line therapy for patient under 70 years old unless special circumstances indicate the prioritization of immediate improvement of symptoms. Our cohort had a relatively higher age of disease onset than the previous study (Tanaka et al., 2013). Therefore, DA might be used less in our cohort than in the previous study.

At baseline, pergolide use was significantly higher in patients with ICB than in patients without ICB. Pergolide use has been reported to be a risk factor for the development of ICB (Weintraub et al., 2006), and findings are consistent with this.

At 2‐year follow‐up, significantly more patients in the group with ICB at baseline had ICB than the group without ICB at baseline. Overall, patients in the ICB persister group were younger, pergolide use than ICB remitters. Younger age and pergolide use have been reported to be risk factors for the development of ICB (Antonini et al., 2017; Weintraub et al., 2006, 2015; Zurowski & O'Brien, 2015). Our findings suggest that these factors may also be related to the persistence of ICB.

D3 receptors may be involved in the pathogenesis of ICB. DAs with preferential affinity for the D3 receptor have strong association with ICB (Moore, Glenmullen, & Mattison, 2014). Pergolide has D3 receptor affinity (Kvernmo, Hartter, & Burger, 2006), and its use has been reported to be risk factors for the development of ICB (Weintraub et al., 2006). Consequently, pergolide might be associated with ICB persistence followed by ICB development. Ergot‐DAs are typically not the first choice of DA because they are risk factors for adverse cardiac valve regurgitation (Schade, Andersohn, Suissa, Haverkamp, & Garbe, 2007). However, Japanese drug information sheets on non‐ergot DAs have physicians instruct the patient not to drive while taking non‐ergot DAs. Accordingly, ergot‐DAs, such as pergolide, may sometimes be the first choice in patients with PD who want to drive. Our region is a relatively rural area in Japan with poor public transportation. ICB persisters were younger than ICB remitters (Table 3); hence, active drivers are more likely to be included in the ICB persister group. Consequently, pergolide might tend to be the medication of choice in younger patients.

The factors previously reported to be associated with ICB remission were reduction in DA use (Avila et al., 2011) and lower DA dosage (Mamikonyan et al., 2008; Sohtaoglu et al., 2010). However, our data did not show any differences in ΔLED‐DA and drug prescription between the ICB persister and ICB remitter groups (Tables 3 and 4). Younger age and pergolide use may potentially be new factors associated with ICB persistence.

The patients who developed ICB in the group of ICB negative at baseline were more often male, and had dyskinesia and zonisamide than patients without ICB. Being male has been reported as a risk factor for the development of ICB (Antonini et al., 2017; Weintraub, Koester, et al., 2010; Weintraub, et al., 2015; Zurowski & O'Brien, 2015). Both L‐DOPA induced dyskinesia (LID), and ICB can be induced by excessive dopaminergic stimulation in the motor or limbic territories of the striatum (Jimenez‐Urbieta et al., 2015; Rocha, Gago, Barbosa, Cavaleiro, & Laranjinha, 2015). Biundo et al. have reported in ALTHEA study, more than half of PD patients with dyskinesia also had ICB. In addition, patients with moderate‐to‐severe dyskinesia had ICB more frequently than patients with only mild dyskinesia (Biundo et al., 2017). DA use and high doses of DA have previously been shown to increase the risk of ICB development (Moore, Glenmullen, et al., 2014; Weintraub, Koester, et al., 2010).

Zonisamide, which is approved as an antiepileptic agent throughout the world, has not been reported as a risk factor for ICB. In 2009, zonisamide was approved for the treatment of PD in Japan. The precise sites of action have remained largely unknown, but zonisamide has multiple actions, including inhibition of sodium channels, T‐type calcium channels, monoamine oxidase‐B activity, activation of dopamine synthesis, and dopamine release (Murata et al., 2015). In a recent study, zonisamide enhanced novelty‐seeking behavior in rats (Uemura, Asano, Hikawa, Yamakado, & Takahashi, 2017). Higher novelty‐seeking traits are known to indicate a greater risk for ICB (Voon et al., 2011). Therefore, we believe that the dopaminergic actions and novelty‐seeking enhancement of zonisamide might be related to the development of ICB in PD.

During the 2‐year follow‐up, rotigotine, entacapone, zonisamide, and istradefylline treatments were significantly more likely to be initiated, or doses increased, in ICB developers than in patients without ICB (Table 4). Although rotigotine has been reported to confer a lower risk compared with other DAs (Garcia‐Ruiz et al., 2014; Moore, Khalaj, et al., 2014; Rizos et al., 2016), rotigotine has D3 receptor affinity, and furthermore, in a post hoc analysis of six open‐label extension studies, 71 of 786 patients (9%) with rotigotine treatment developed ICB (Antonini et al., 2016). Hence, rotigotine could be a risk factor for the development of ICB. An association between entacapone and ICB has not been reported to date. Entacapone, a catechol‐O‐methyltransferase (COMT) inhibitor, reduces peripheral catabolism of L‐DOPA and improves its effective delivery to the brain, and has subsequent clinical effects, in PD patients with motor fluctuations (Lees, 2008). Concurrent use of L‐DOPA and DAs, and high dose of L‐DOPA have been linked to ICB such as DDS (Voon et al., 2011; Weintraub et al., 2015). Changes in COMT expression have also been associated with impulsivity (Vevera et al., 2009). Therefore, we speculate that entacapone seems to be involved in the development of ICB.

The association between istradefylline and ICB has not been reported. Istradefylline, a selective adenosine A_2A_ receptor antagonist, has nondopaminergic effects because it lacks effects on dopamine receptors and dopamine‐metabolizing enzymes (Mizuno & Kondo, 2013). Istradefylline improves motor function, without worsening LID, in animal models. A clinical trial has shown that istradefylline is effective in improving motor symptoms in PD patients (Mizuno & Kondo, 2013) and it was approved for the treatment of wearing off under L‐DOPA co‐administration in PD patients in Japan in 2013. Adenosine A_2A_ receptors are abundant in the striatum and other nuclei of the basal ganglia, as well as in the nucleus accumbens (Rosin, Hettinger, Lee, & Linden, 2003), which plays a key role in the development of DDS (Evans et al., 2006), and adenosine A_2A_ receptors modulate the reward and reinforcement pathways (Filip, Zaniewska, Frankowska, Wydra, & Fuxe, 2012). Therefore, we hypothesize that the adenosine A_2A_ receptor antagonist istradefylline could cause excitatory stimulation in the mesolimbic area, leading to the development of ICB.

Entacapone monotherapy became available in Japan from December 2015. Until then, entacapone had been used in combination with DRT, with zonisamide and istradefylline only available with DRT at the current time. Therefore, entacapone treatment may tend to be initiated earlier than zonisamide and istradefylline. In addition, DRT use tends to be difficult in patients with ICB, and consequently, in our practice nondopaminergic drugs, such as zonisamide and istradefylline, tend to be used in patients with ICB. Further investigation is needed to confirm the association between ICB and entacapone, zonisamide, and istradefylline.

In the group without ICB at baseline, pramipexole ER were more frequently discontinued or the dose reduced in ICB developers than in patients who had not developed ICB during the 2‐year follow‐up. Two of the three patients in the ICB developer group had reduced their doses of pramipexole ER to mitigate ICB, so the reduction in pramipexole ER seems to be a result of the development of ICB rather than the cause.

There are some limitations of this study. First, this was a retrospective observational study, so our conclusions about the factors associated with the development of ICB may be tenuous. Second, the QUIP assessment was conducted by the patients or their caregivers and has some delicate questions, such as regarding sexual activity, so they may feel uncomfortable completing it honestly (Zurowski & O'Brien, 2015). Some people feel ashamed or embarrassed about these behaviors and would not broach the subject by direct screening. Therefore, the prevalence of ICB in our study may underestimate the actual prevalence. Third, we were not able to conduct multivariate analyses with respect to the associated factors due to the small number of patients. Finally, this study was conducted in a single center and our patients may not represent the general PD population and the selection bias was not removed in each group. Prospective and larger sized studies are needed to confirm the association between ICB persistence or development and associated factors.

## 5 CONCLUSIONS

This study suggests that younger age and pergolide use may be potential new factors associated with ICB persistence, and further, entacapone, zonisamide, and istradefylline may be new factors associated with the development of ICB. Drug profiles and medication practices in Japan may explain the association of these factors with ICB. Physicians should pay careful attention to these potential factors when treating patients with PD to help avoid the development or persistence of ICB.

## CONFLICT OF INTEREST

The authors declare that they have no conflict of interests nor any funding sources to declare.

## AUTHOR CONTRIBUTIONS

T.K, T.U, and M.T. contributed to the design of the study; T.U. and M.T. supervised the study; T.K, T.U, R.H. and M.T. collected the data; T.K. drafted the manuscript; T.U. and M.T. critically reviewed the manuscript.

## References

[brb31036-bib-0001] Antonini, A. , Barone, P. , Bonuccelli, U. , Annoni, K. , Asgharnejad, M. , & Stanzione, P. (2017). ICARUS study: Prevalence and clinical features of impulse control disorders in Parkinson's disease. Journal of Neurology, Neurosurgery and Psychiatry, 88(4), 317–324. 10.1136/jnnp-2016-315277 28315845

[brb31036-bib-0002] Antonini, A. , Chaudhuri, K. R. , Boroojerdi, B. , Asgharnejad, M. , Bauer, L. , Grieger, F. , & Weintraub, D. (2016). Impulse control disorder related behaviours during long‐term rotigotine treatment: A post hoc analysis. European Journal of Neurology, 23(10), 1556–1565. 10.1111/ene.13078 27425586 PMC5096013

[brb31036-bib-0003] Avila, A. , Cardona, X. , Martin‐Baranera, M. , Bello, J. , & Sastre, F. (2011). Impulsive and compulsive behaviors in Parkinson's disease: A one‐year follow‐up study. Journal of the Neurological Sciences, 310(1–2), 197–201. 10.1016/j.jns.2011.05.044 21683375

[brb31036-bib-0004] Bastiaens, J. , Dorfman, B. J. , Christos, P. J. , & Nirenberg, M. J. (2013). Prospective cohort study of impulse control disorders in Parkinson's disease. Movement Disorders, 28(3), 327–333. 10.1002/mds.25291 23283708 PMC3894820

[brb31036-bib-0005] Biundo, R. , Weis, L. , Abbruzzese, G. , Calandra‐Buonaura, G. , Cortelli, P. , Jori, M. C. , … Antonini, A. (2017). Impulse control disorders in advanced Parkinson's disease with dyskinesia: The ALTHEA study. Movement Disorders, 32(11), 1557–1565. 10.1002/mds.27181 28960475

[brb31036-bib-0006] Callesen, M. B. , Weintraub, D. , Damholdt, M. F. , & Moller, A. (2014). Impulsive and compulsive behaviors among Danish patients with Parkinson's disease: Prevalence, depression, and personality. Parkinsonism & Related Disorders, 20(1), 22–26. 10.1016/j.parkreldis.2013.09.006 24090948

[brb31036-bib-0007] Cera, N. , Bifolchetti, S. , Martinotti, G. , Gambi, F. , Sepede, G. , Onofrj, M. , … Thomas, A. (2014). Amantadine and cognitive flexibility: Decision making in Parkinson's patients with severe pathological gambling and other impulse control disorders. Neuropsychiatric Disease and Treatment, 10, 1093–1101. 10.2147/NDT.S54423 24971012 PMC4069151

[brb31036-bib-0008] Chiang, H. L. , Huang, Y. S. , Chen, S. T. , & Wu, Y. R. (2012). Are there ethnic differences in impulsive/compulsive behaviors in Parkinson's disease? European Journal of Neurology, 19(3), 494–500. 10.1111/j.1468-1331.2011.03571.x 22040282

[brb31036-bib-0009] Christenson, G. A. , Faber, R. J. , de Zwaan, M. , Raymond, N. C. , Specker, S. M. , Ekern, M. D. , … Mitchell, J. E. (1994). Compulsive buying: Descriptive characteristics and psychiatric comorbidity. Journal of Clinical Psychiatry, 55(1), 5–11. 8294395

[brb31036-bib-0010] Comings, D. E. , & Blum, K. (2000). Reward deficiency syndrome: Genetic aspects of behavioral disorders. Progress in Brain Research, 126, 325–341. 10.1016/S0079-6123(00)26022-6 11105655

[brb31036-bib-0011] Erga, A. H. , Alves, G. , Larsen, J. P. , Tysnes, O. B. , & Pedersen, K. F. (2017). Impulsive and compulsive behaviors in Parkinson's disease: The Norwegian ParkWest Study. Journal of Parkinson's Disease, 7(1), 183–191. 10.3233/JPD-160977 PMC5302042 27911342

[brb31036-bib-0012] Evans, A. H. , Pavese, N. , Lawrence, A. D. , Tai, Y. F. , Appel, S. , Doder, M. , … Piccini, P. (2006). Compulsive drug use linked to sensitized ventral striatal dopamine transmission. Annals of Neurology, 59(5), 852–858. 10.1002/ana.20822 16557571

[brb31036-bib-0013] Fan, W. , Ding, H. , Ma, J. , & Chan, P. (2009). Impulse control disorders in Parkinson's disease in a Chinese population. Neuroscience Letters, 465(1), 6–9. 10.1016/j.neulet.2009.06.074 19560522

[brb31036-bib-0014] Filip, M. , Zaniewska, M. , Frankowska, M. , Wydra, K. , & Fuxe, K. (2012). The importance of the adenosine A(2A) receptor‐dopamine D(2) receptor interaction in drug addiction. Current Medicinal Chemistry, 19(3), 317–355. 22335511 10.2174/092986712803414231

[brb31036-bib-0015] Garcia‐Ruiz, P. J. , Martinez Castrillo, J. C. , Alonso‐Canovas, A. , Herranz Barcenas, A. , Vela, L. , Sanchez Alonso, P. , … Mahillo Fernandez, I. (2014). Impulse control disorder in patients with Parkinson's disease under dopamine agonist therapy: A multicentre study. Journal of Neurology, Neurosurgery and Psychiatry, 85(8), 840–844. 10.1136/jnnp-2013-306787 24434037

[brb31036-bib-0016] Holsinger, T. , Deveau, J. , Boustani, M. , & Williams, Jr, J. W. (2007). Does this patient have dementia? JAMA, 297(21), 2391–2404. 10.1001/jama.297.21.2391 17551132

[brb31036-bib-0017] Hughes, A. J. , Daniel, S. E. , Kilford, L. , & Lees, A. J. (1992). Accuracy of clinical diagnosis of idiopathic Parkinson's disease: A clinico‐pathological study of 100 cases. Journal of Neurology, Neurosurgery and Psychiatry, 55(3), 181–184. 10.1136/jnnp.55.3.181 PMC1014720 1564476

[brb31036-bib-0018] Ibanez, A. , Blanco, C. , Perez de Castro, I. , Fernandez‐Piqueras, J. , & Saiz‐Ruiz, J. (2003). Genetics of pathological gambling. Journal of Gambling Studies, 19(1), 11–22. 12635538 10.1023/a:1021271029163

[brb31036-bib-0019] Jimenez‐Urbieta, H. , Gago, B. , de la Riva, P. , Delgado‐Alvarado, M. , Marin, C. , & Rodriguez‐Oroz, M. C. (2015). Dyskinesias and impulse control disorders in Parkinson's disease: From pathogenesis to potential therapeutic approaches. Neuroscience and Biobehavioral Reviews, 56, 294–314. 10.1016/j.neubiorev.2015.07.010 26216865

[brb31036-bib-0020] Kraemmer, J. , Smith, K. , Weintraub, D. , Guillemot, V. , Nalls, M. A. , Cormier‐Dequaire, F. , … Corvol, J. C. (2016). Clinical‐genetic model predicts incident impulse control disorders in Parkinson's disease. Journal of Neurology, Neurosurgery and Psychiatry, 87(10), 1106–1111. 10.1136/jnnp-2015-312848 PMC5098340 27076492

[brb31036-bib-0021] Kvernmo, T. , Hartter, S. , & Burger, E. (2006). A review of the receptor‐binding and pharmacokinetic properties of dopamine agonists. Clinical Therapeutics, 28(8), 1065–1078. 10.1016/j.clinthera.2006.08.004 16982285

[brb31036-bib-0022] Le Foll, B. , Gallo, A. , Le Strat, Y. , Lu, L. , & Gorwood, P. (2009). Genetics of dopamine receptors and drug addiction: A comprehensive review. Behavioural Pharmacology, 20(1), 1–17. 10.1097/FBP.0b013e3283242f05 19179847

[brb31036-bib-0023] Lee, J. Y. , Jeon, B. S. , Kim, H. J. , & Park, S. S. (2012). Genetic variant of HTR2A associates with risk of impulse control and repetitive behaviors in Parkinson's disease. Parkinsonism & Related Disorders, 18(1), 76–78. 10.1016/j.parkreldis.2011.08.009 21900033

[brb31036-bib-0024] Lee, J. Y. , Lee, E. K. , Park, S. S. , Lim, J. Y. , Kim, H. J. , Kim, J. S. , & Jeon, B. S. (2009). Association of DRD3 and GRIN2B with impulse control and related behaviors in Parkinson's disease. Movement Disorders, 24(12), 1803–1810. 10.1002/mds.22678 19562769

[brb31036-bib-0025] Lees, A. J. (2008). Evidence‐based efficacy comparison of tolcapone and entacapone as adjunctive therapy in Parkinson's disease. CNS Neuroscience & Therapeutics, 14(1), 83–93. 10.1111/j.1527-3458.2007.00035.x 18482101 PMC6494077

[brb31036-bib-0026] Lim, S. Y. , Tan, Z. K. , Ngam, P. I. , Lor, T. L. , Mohamed, H. , Schee, J. P. , … Soh, P. C. (2011). Impulsive–compulsive behaviors are common in Asian Parkinson's disease patients: Assessment using the QUIP. Parkinsonism & Related Disorders, 17(10), 761–764. 10.1016/j.parkreldis.2011.07.009 21839665

[brb31036-bib-0027] Mamikonyan, E. , Siderowf, A. D. , Duda, J. E. , Potenza, M. N. , Horn, S. , Stern, M. B. , & Weintraub, D. (2008). Long‐term follow‐up of impulse control disorders in Parkinson's disease. Movement Disorders, 23(1), 75–80. 10.1002/mds.21770 17960796 PMC2651355

[brb31036-bib-0028] Mizuno, Y. , & Kondo, T. ; Japanese Istradefylline Study Group . (2013). Adenosine A_2A_ receptor antagonist istradefylline reduces daily OFF time in Parkinson's disease. Movement Disorders, 28(8), 1138–1141. 10.1002/mds.25418 23483627 PMC3842830

[brb31036-bib-0029] Moore, T. J. , Glenmullen, J. , & Mattison, D. R. (2014). Reports of pathological gambling, hypersexuality, and compulsive shopping associated with dopamine receptor agonist drugs. JAMA Internal Medicine, 174(12), 1930–1933. 10.1001/jamainternmed.2014.5262 25329919

[brb31036-bib-0030] Moore, S. M. , Khalaj, A. J. , Kumar, S. , Winchester, Z. , Yoon, J. , Yoo, T. , … Tiwari‐Woodruff, S. K. (2014). Multiple functional therapeutic effects of the estrogen receptor beta agonist indazole‐Cl in a mouse model of multiple sclerosis. Proceedings of the National Academy of Sciences of the United States of America, 111(50), 18061–18066. 10.1073/pnas.1411294111 25453074 PMC4273334

[brb31036-bib-0031] Murata, M. , Hasegawa, K. , Kanazawa, I. , Fukasaka, J. , Kochi, K. , & Shimazu, R. ; Japan Zonisamide on PD Study Group . (2015). Zonisamide improves wearing‐off in Parkinson's disease: A randomized, double‐blind study. Movement Disorders, 30(10), 1343–1350. 10.1002/mds.26286 26094993

[brb31036-bib-0032] Rizos, A. , Sauerbier, A. , Antonini, A. , Weintraub, D. , Martinez‐Martin, P. , Kessel, B. , … EUROPAR and the IPMDS Non‐Motor‐PD‐Study Group . (2016). A European multicentre survey of impulse control behaviours in Parkinson's disease patients treated with short‐ and long‐acting dopamine agonists. European Journal of Neurology, 23(8), 1255–1261. 10.1111/ene.13034 27170229

[brb31036-bib-0033] Rocha, B. S. , Gago, B. , Barbosa, R. M. , Cavaleiro, C. , & Laranjinha, J. (2015). Ethyl nitrite is produced in the human stomach from dietary nitrate and ethanol, releasing nitric oxide at physiological pH: Potential impact on gastric motility. Free Radical Biology and Medicine, 82, 160–166. 10.1016/j.freeradbiomed.2015.01.021 25645954

[brb31036-bib-0034] Rodriguez‐Violante, M. , Gonzalez‐Latapi, P. , Cervantes‐Arriaga, A. , Camacho‐Ordonez, A. , & Weintraub, D. (2014). Impulse control and related disorders in Mexican Parkinson's disease patients. Parkinsonism & Related Disorders, 20(8), 907–910. 10.1016/j.parkreldis.2014.05.014 24935236

[brb31036-bib-0035] Rosin, D. L. , Hettinger, B. D. , Lee, A. , & Linden, J. (2003). Anatomy of adenosine A_2A_ receptors in brain: Morphological substrates for integration of striatal function. Neurology, 61(11 Suppl 6), S12–S18. 10.1212/01.wnl.0000095205.33940.99 14663003

[brb31036-bib-0036] Schade, R. , Andersohn, F. , Suissa, S. , Haverkamp, W. , & Garbe, E. (2007). Dopamine agonists and the risk of cardiac‐valve regurgitation. New England Journal of Medicine, 356(1), 29–38. 10.1056/NEJMoa062222 17202453

[brb31036-bib-0037] Sharma, A. , Goyal, V. , Behari, M. , Srivastva, A. , Shukla, G. , & Vibha, D. (2015). Impulse control disorders and related behaviours (ICD‐RBs) in Parkinson's disease patients: Assessment using “Questionnaire for impulsive–compulsive disorders in Parkinson's disease” (QUIP). Annals of Indian Academy of Neurology, 18(1), 49–59. 10.4103/0972-2327.144311 25745311 PMC4350214

[brb31036-bib-0038] Siri, C. , Cilia, R. , Reali, E. , Pozzi, B. , Cereda, E. , Colombo, A. , … Pezzoli, G. (2015). Long‐term cognitive follow‐up of Parkinson's disease patients with impulse control disorders. Movement Disorders, 30(5), 696–704. 10.1002/mds.26160 25757654

[brb31036-bib-0039] Smith, K. M. , Xie, S. X. , & Weintraub, D. (2016). Incident impulse control disorder symptoms and dopamine transporter imaging in Parkinson disease. Journal of Neurology, Neurosurgery and Psychiatry, 87(8), 864–870. 10.1136/jnnp-2015-311827 PMC4854795 26534930

[brb31036-bib-0040] Sohtaoglu, M. , Demiray, D. Y. , Kenangil, G. , Ozekmekci, S. , & Erginoz, E. (2010). Long term follow‐up of Parkinson's disease patients with impulse control disorders. Parkinsonism & Related Disorders, 16(5), 334–337. 10.1016/j.parkreldis.2010.02.006 20223696

[brb31036-bib-0041] Tanaka, K. , Wada‐Isoe, K. , Nakashita, S. , Yamamoto, M. , & Nakashima, K. (2013). Impulsive compulsive behaviors in Japanese Parkinson's disease patients and utility of the Japanese version of the Questionnaire for Impulsive–Compulsive Disorders in Parkinson's disease. Journal of the Neurological Sciences, 331(1–2), 76–80. 10.1016/j.jns.2013.05.013 23735774

[brb31036-bib-0042] Tomlinson, C. L. , Stowe, R. , Patel, S. , Rick, C. , Gray, R. , & Clarke, C. E. (2010). Systematic review of levodopa dose equivalency reporting in Parkinson's disease. Movement Disorders, 25(15), 2649–2653. 10.1002/mds.23429 21069833

[brb31036-bib-0043] Uemura, M. T. , Asano, T. , Hikawa, R. , Yamakado, H. , & Takahashi, R. (2017). Zonisamide inhibits monoamine oxidase and enhances motor performance and social activity. Neuroscience Research, 124, 25–32. 10.1016/j.neures.2017.05.008 28624436

[brb31036-bib-0044] Vela, L. , Martinez Castrillo, J. C. , Garcia Ruiz, P. , Gasca‐Salas, C. , Macias Macias, Y. , Perez Fernandez, E. , … Marasescu, R. (2016). The high prevalence of impulse control behaviors in patients with early‐onset Parkinson's disease: A cross‐sectional multicenter study. Journal of the Neurological Sciences, 368, 150–154. 10.1016/j.jns.2016.07.003 27538621

[brb31036-bib-0045] Vevera, J. , Stopkova, R. , Bes, M. , Albrecht, T. , Papezova, H. , Zukov, I. , … Stopka, P. (2009). COMT polymorphisms in impulsively violent offenders with antisocial personality disorder. Neuro Endocrinology Letters, 30(6), 753–756. 20038933

[brb31036-bib-0046] Voon, V. , & Fox, S. H. (2007). Medication‐related impulse control and repetitive behaviors in Parkinson disease. Archives of Neurology, 64(8), 1089–1096. 10.1001/archneur.64.8.1089 17698698

[brb31036-bib-0047] Voon, V. , Sohr, M. , Lang, A. E. , Potenza, M. N. , Siderowf, A. D. , Whetteckey, J. , … Stacy, M. (2011). Impulse control disorders in Parkinson disease: A multicenter case–control study. Annals of Neurology, 69(6), 986–996. 10.1002/ana.22356 21416496

[brb31036-bib-0048] Wang, X. P. , Wei, M. , & Xiao, Q. (2016). A survey of impulse control disorders in Parkinson's disease patients in Shanghai area and literature review. Translational Neurodegeneration, 5, 4 10.1186/s40035-016-0051-7 26893825 PMC4758007

[brb31036-bib-0049] Weintraub, D. , David, A. S. , Evans, A. H. , Grant, J. E. , & Stacy, M. (2015). Clinical spectrum of impulse control disorders in Parkinson's disease. Movement Disorders, 30(2), 121–127. 10.1002/mds.26016 25370355

[brb31036-bib-0050] Weintraub, D. , Hoops, S. , Shea, J. A. , Lyons, K. E. , Pahwa, R. , Driver‐Dunckley, E. D. , … Voon, V. (2009). Validation of the questionnaire for impulsive–compulsive disorders in Parkinson's disease. Movement Disorders, 24(10), 1461–1467. 10.1002/mds.22571 19452562 PMC2848971

[brb31036-bib-0051] Weintraub, D. , Koester, J. , Potenza, M. N. , Siderowf, A. D. , Stacy, M. , Voon, V. , … Lang, A. E. (2010). Impulse control disorders in Parkinson disease: A cross‐sectional study of 3090 patients. Archives of Neurology, 67(5), 589–595. 10.1001/archneurol.2010.65 20457959

[brb31036-bib-0052] Weintraub, D. , Papay, K. , & Siderowf, A. ; Parkinson's Progression Markers Initiative . (2013). Screening for impulse control symptoms in patients with de novo Parkinson disease: A case–control study. Neurology, 80(2), 176–180. 10.1212/WNL.0b013e31827b915c 23296128 PMC3589192

[brb31036-bib-0053] Weintraub, D. , Siderowf, A. D. , Potenza, M. N. , Goveas, J. , Morales, K. H. , Duda, J. E. , … Stern, M. B. (2006). Association of dopamine agonist use with impulse control disorders in Parkinson disease. Archives of Neurology, 63(7), 969–973. 10.1001/archneur.63.7.969 16831966 PMC1761054

[brb31036-bib-0054] Weintraub, D. , Sohr, M. , Potenza, M. N. , Siderowf, A. D. , Stacy, M. , Voon, V. , … Lang, A. E. (2010). Amantadine use associated with impulse control disorders in Parkinson disease in cross‐sectional study. Annals of Neurology, 68(6), 963–968. 10.1002/ana.22164 21154480

[brb31036-bib-0055] Zainal Abidin, S. , Tan, E. L. , Chan, S. C. , Jaafar, A. , Lee, A. X. , Abd Hamid, M. H. , … Mohamed Ibrahim, N. (2015). DRD and GRIN2B polymorphisms and their association with the development of impulse control behaviour among Malaysian Parkinson's disease patients. BMC Neurology, 15, 59 10.1186/s12883-015-0316-2 25896831 PMC4417293

[brb31036-bib-0056] Zurowski, M. , & O'Brien, J. D. (2015). Developments in impulse control behaviours of Parkinson's disease. Current Opinion in Neurology, 28(4), 387–392. 10.1097/WCO.0000000000000209 26110803

